# Loss of reflex tearing after maxillary orthognathic surgery: a report of two cases

**DOI:** 10.1186/1471-2415-14-37

**Published:** 2014-03-28

**Authors:** Sunah Kang, Sun Young Jang, Areum Lee, Jae Woo Jang

**Affiliations:** 1 Department of Ophthalmology, Kim’s Eye Hospital, Myung-Gok Eye Research Institute, Konyang University College of Medicine, Youngdeungpo 4th 156, Youngdeungpo-gu 150-034, Seoul, Korea; 2 Department of Ophthalmology, Soonchunhyang Bucheon Hospital, Soonchunhyang University College of Medicine, Bucheon, Korea; 3 Department of Radiology, Soonchunhyang Bucheon Hospital, Soonchunhyang University College of Medicine, Bucheon, Korea

**Keywords:** Reflex tearing loss, Orthognathic surgery, Complication

## Abstract

**Background:**

Few reports have described the ophthalmic complications that occur after maxillary orthognathic surgery. Since cases of decreased reflex tearing after maxillary orthognathic surgery are extremely rare, we describe 2 cases of loss of reflex tearing after maxillary orthognathic surgery.

**Case presentation:**

Two Asian women, an 18-year-old and a 32-year-old, suffered from unilateral dryness and irritation caused by maxillary orthognathic surgery. In both patients, Schirmer test (II) showed reduced reflex tearing in 1 eye. Computed tomography showed that the pterygoid plate had been fractured in both patients.

**Conclusions:**

The pterygopalatine ganglion and its associated fibers in the pterygopalatine fossa may be injured during Le Fort osteotomy.

## Background

Few reports have described the ophthalmic complications that can potentially occur after maxillary orthognathic surgery [1]. The Le Fort I and Le Fort III osteotomy techniques are used during maxillary orthognathic surgery. Le Fort I osteotomy frees the maxilla from the remainder of the facial skeleton through a transmaxillary fracture that includes the maxillary sinuses, lateral margin of the nasal fossa, and nasal septum. Subsequently, the maxilla can be moved upward, downward, anteriorly, or posteriorly. Le Fort III osteotomy enables the separation of the entire midfacial complex from the base of the skull, allowing movement of the midface independent of the cranium and mandible [2]. Osteotomies in Le Fort III are made through the frontozygomatic suture, floor of the orbit, and the nasion using a reciprocating saw [3]. Incidents of vision loss, extraocular muscle dysfunction (3rd/6th cranial nerve palsy), neuroparalytic keratitis, lacrimal drainage obstruction, and keratitis sicca following these procedures have been reported [1,4-6]. One possible etiology for keratits sicca is that untoward fractures from maxillary orthognathic surgery [1]. Since cases of decreased reflex tearing after maxillary orthognathic surgery are rare, we present two case reports along with the findings of radiological analysis and a review of the literature. To our best knowledge, this is the first report describing the loss of reflex tearing after maxillary orthognathic surgery in Asian patients.

## Case presentation

### Case 1

The first patient was an 18-year-old woman who presented with the chief complaint of dryness of the right eye. The patient had undergone maxillary orthognathic surgery at a dental clinic 1 month before presentation. She developed symptoms of decreased tearing in the right eye 1 week after surgery and reported that only the left side of her nose became congested when she developed a cold.

**Figure 1:**
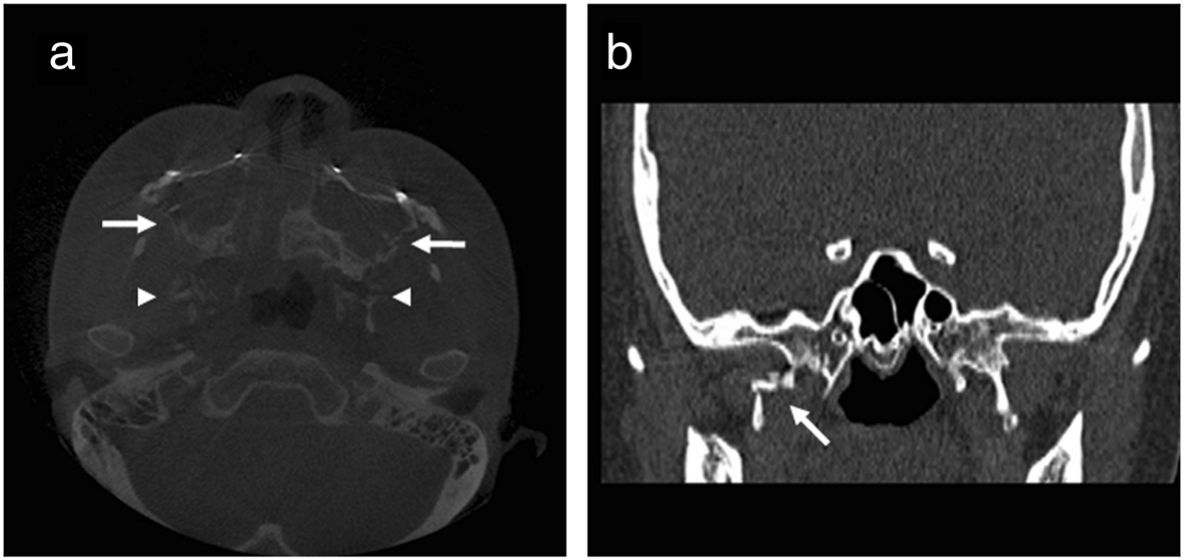
**A computed tomography (CT) scan of case 1. (a)** Axial view, note the fractures in the lateral walls of both maxillary sinuses (arrows) and both pterygoid plates (arrowheads). **(b)** coronal view, note the fracture in the right pterygoid plate (arrows).

At the time of her visit, the patient’s corrected vision was 20/20 for both eyes. No relative afferent pupillary defect was observed. Upon slit-lamp examination, punctuated epithelial erosion was observed on the cornea of the right eye. Hypoesthesia to light touch in the right malar area, which is the domain of the zygomaticofacial nerve, was also confirmed. The patient’s tear film breakup time was 5 s in the right eye and 7 s in the left eye. The findings of Schirmer I test were 5 mm for the right eye and 10 mm for the left eye. The results of the Schirmer II test, which measures the degree of reflex tearing by irritating the nasal cavities, were 4 mm for the right eye and 12 mm for the left. The computed tomographic (CT) image obtained at the dental clinic immediately after the surgery confirmed a Le Fort I fracture across both pterygoid plates and both maxillary sinuses. Although pterygoid plate fracture were visualized bilaterally, the severity of the fracture was much worse on the right side in the present case (Figure 1).

To improve the patient’s symptoms, an ophthalmic ointment and preservative-free artificial tears were prescribed for the right eye, and her progress was monitored. The patient continued to experience decreased tearing in the right eye, but overall, the symptoms had improved 6 months after the surgery. After treatment, the Schirmer I test measurements were 5 mm for the right eye and 10 mm for the left eye, and the Schirmer II test measurements were 7 mm for the right eye and 12 mm for the left eye.

### Case 2

The second patient was a 32-year-old woman who presented with the chief complaints of decreased tearing in the right eye and excessive tearing in the left eye. The patient had undergone maxillary orthognathic surgery at a dental clinic 6 months before presentation.

**Figure 2:**
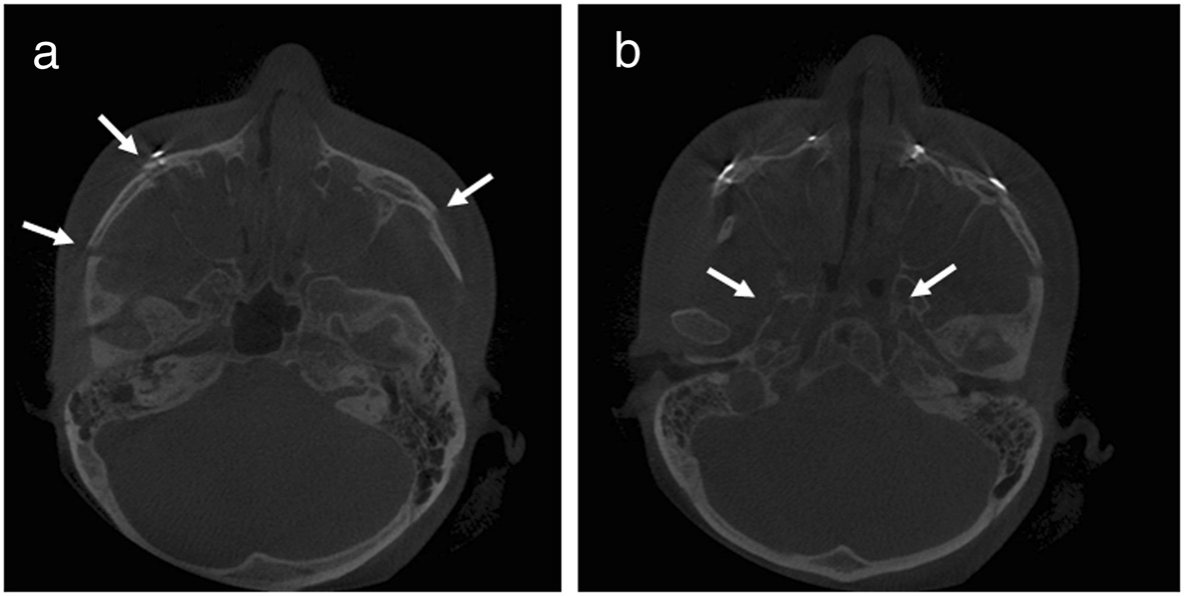
**A CT scan of case 2. (a)** Axial view, fractures in the medial and lateral walls of both maxillary sinuses, both zygomatic arches (arrows) and **(b)** both pterygoid plates (arrows).

At the time of her visit, the patient’s vision was 20/20 in both eyes. Upon slit-lamp examination, blepharitis was observed. The height of the tear meniscus was 0.5 mm for the right eye and 1 mm for the left eye. The Schirmer I test measurements were 7 mm for the right eye and 9 mm for the left eye, and the Schirmer II test measurements were 5 mm for the right eye and 11 mm for the left eye. A syringe test revealed normal results for the right eye but regurgitation from the opposite punctum in the left eye. Dacryocystography was performed, revealing complete obstruction of the nasolacrimal duct at the junction of the sac and duct in the left eye. In the CT image obtained at the dental clinic immediately after surgery, a Le Fort III fracture was observed across both pterygoid plates, the lateral and distal walls of both maxillary sinuses, and both zygomatic arches (Figure 2). The patient underwent endonasal dacryocystorhinostomy (DCR) in the left eye 7 months after the maxillary surgery. The DCR restored normal tear function in the left eye. Preservative-free artificial tears were prescribed for the right eye. These treatments improved the patient’s complaints of dry eye. However, the patient still complained of decreased tearing in the right eye 8 months after the initial visit to our clinic.

## Conclusions

Decreased reflex tearing after maxillary orthognathic surgery is rare. To the best of our knowledge, there are only 3 cases in the current literature. In 1976, Tomasetti et al. [5] first reported a case of decreased tearing after Le Fort I osteotomy. The authors suggested that the decrease in tearing was related to a pterygoid plate fracture during surgery. In 1993, Lanigan et al. [4] used CT images to confirm fractures in both the pterygoid plate and the posterolateral wall of the right maxillary sinus, which resulted in decreased tearing after surgery. The authors believed that these fractures extended to the pterygopalatine fossa, thereby damaging the pterygopalatine ganglion, which is one of the nervous structures involved with lacrimation [1]. Although decreased reflex tearing after maxillary orthognathic surgery has rarely been reported, decreased tearing after surgical removal of juvenile nasopharyngeal angiofibromas has been well documented. In such cases, damage to the pterygopalatine ganglion during surgery results in reduced reflex tearing [7-11]. During the pterygomaxillary dysjunction and maxillary downfracture associated with a LeFort I osteotomy, high-level pterygoid plate fractures can occur near the base of the skull and can disrupt the contents of the pterygopalatine fossa. Aberrant fractures can also extend to the base of the skull, to areas such as the foramen lacerum, pterygoid canal, and sphenoid sinus, or to the orbit including the inferior orbital fissure and optic canal. Due to the complexity of the nerve supply involved with lacrimation there is a potential for damage to have occurred at multiple levels resulting in a dry eye and not just from an injury to the pterygopalatine ganglion. Nerves can be immobilized between bone fragments or fracture gaps, leading to nerve damage in the pterygopalatine ganglion, postganglionic parasympathetic fibers, and the zygomatic branch of the maxillary nerve. In particular, postganglionic parasympathetic fibers are nonmyelinated fibers and are more vulnerable to damage than myelinated fibers; thus, these fibers can be selectively damaged under equal amounts of external force [5].

Other possible explanations for a loss of reflex tearing include a spread of the postsurgical inflammatory response to the pterygopalatine ganglion region, causing damage to nearby tissue or to the descending palatine or maxillary arteries, which in turn causes ischemic damage to the pterygopalatine ganglion [4]. Nasolacrimal duct obstruction after maxillary orthognathic surgery has previously been reported by Jang et al. [6] On the basis of the clinical and radiologic findings, the authors have concluded that nasolacrimal duct obstruction after orthognathic surgery may develop because of a postsurgical inflammatory response to indirect damage of the nasolacrimal duct at the time of surgery.

Chrcanovic and Custódio mentioned that a thorough understanding of the anatomy of the pterymaxillary junction, the forces induced during surgery, and the use of appropriate instrumentation can reduce the incidence and severity of complications associated with Le Fort I osteotomy [12]. In the present study, we have reported that decreased reflex tearing could arise as a result of a high-level pterygoid plate fracture, or an aberrant fracture line extending high up along the posterior wall of the maxilla, that injured neural structures within the pterygopalatine fossa in conjunction either with the pterygomaxillary dysjunction with an osteotome or the maxillary downfracture. The best way to minimize the occurrence of this type of complication would be to utilize a method of achieving the pterygomaxillary separation that minimizes the chance of a high-level pterygoid plate fracture, such as the use of a micro-oscillating saw [13]. Although pterygoid plate fractures were visualized bilaterally in both cases after the Le Fort osteotomies, the severity of the fractures was much worse on the side where the patient developed a dry eye as a postoperative complication. The symptoms improved over time with conservative treatment in both patients. Nonetheless, it is important to educate patients adequately on the ophthalmic complications that can occur after maxillary orthognathic surgery.

Since all subjects in the present study were referred to our center from other clinics for evaluation and management of an ocular symptom, there is a lack of information regarding the surgical techniques used by the two different surgeons, and this is a limitation of our report. A second limitation was we could not get more detailed images, like 3-D reconstruction of CT scans, to better visualize the possible disruption of the pterygopalatine fossa. However, we still found that the loss of reflex tearing can occur after maxillary orthognathic surgery. Furthermore, we found that this condition can recover spontaneously.

## Consent

Written informed consent was obtained from both of the patients for publication of this case report and any accompanying images. A copy of the written consent is available for review by the Editor of this journal.

## Abbreviations

CT: Computed tomography; DCR: Dacryocystorhinostomy.

## Competing interests

The authors declare that they have no competing interests.

## Authors’ contributions

JWJ and SYJ was responsible for the conception and design of the project. SYJ and AL were responsible for acquisition and interpretation of data. SAK was responsible for drafting the article. JWJ and SYJ revised the manuscript critically for important intellectual content. All authors approved the final version to be published.

## Pre-publication history

The pre-publication history for this paper can be accessed here:

http://www.biomedcentral.com/1471-2415/14/37/prepub
